# To mix or not to mix? Medicolegal implications of mixed components in total hip arthroplasty

**DOI:** 10.1080/17453674.2020.1822066

**Published:** 2020-09-23

**Authors:** Rinne M Peters, Jantina T Hiemstra, Wierd P Zijlstra, Sjoerd K Bulstra, Martin Stevens

**Affiliations:** a Department of Orthopedic Surgery, Medical Center Leeuwarden;; b Department of Orthopedic Surgery, University of Groningen, University Medical Center Groningen;; c Hausfeld Advocaten, Amsterdam;; d Department of Private law, University of Groningen, The Netherlands

About 300 different hip prostheses promoted by a multitude of distributors are available on the European market. Most total hip arthroplasties (THAs) are assembled from components produced by the same manufacturer (non-mixed THAs), yet certain situations require a combination of components from different manufacturers within a single hip prosthesis (mixed THAs). Despite it being against manufacturers’ guidelines (Smith & Nephew 2013, Link 2018), orthopedic surgeons who do this are encouraged by clinical results that are comparable to and sometimes even superior to those obtained without mixed components (Tucker et al. 2015, Peters et al. 2016, Taylor et al. 2018). This mixing and matching is common clinical practice. The question does remain as to whether it is allowable by law. In this annotation paper we assess the legality of mixed THAs based on European law.

## Mix and match: clinical perspective

Mixed prostheses are defined as THAs (stem, head, and cup) comprising components made by different manufactures. With a reported prevalence of 11%, 24%, and 15% in the Netherlands, New Zealand, and England and Wales, respectively, mixing and matching is common clinical practice (Tucker et al. 2015, Peters et al. 2016, Taylor et al. 2018). Based on these national joint registry studies, it was demonstrated that mixed THAs yield at least comparable and for certain combinations even better outcomes than THAs with components from the same manufacturer (Tucker et al. 2015, Peters et al. 2016, Taylor et al. 2018).

The concept of mixed THA refers to both fixed (trunnion/taper) and mobile (head/cup) combinations as well as hard-on-soft and hard-on-hard bearings. A distinction should additionally be made between primary and revision procedures. An argument for the use of mixed components in primary THA could be the need for a dual mobility cup in case of high risk of instability. Other arguments could be altered anatomy (e.g., developmental dysplasia of the hip), patient characteristics (e.g., frail elderly patients requiring cemented stems), and high-risk patients (e.g., prior lumbar spine fusion with irradiated pelvis). In revision arthroplasty, combining components from different manufacturers could be considered in order to prevent additional patient morbidity (e.g., leaving a well-fixed stem from another company in situ during a cup revision) (Mueller et al. 2018), or to optimize component placement performed by surgeons with extensive clinical experience. This is all in the best interest of the patient.

Hard-on-soft mixing and matching across the femoral head and acetabular component (mobile bearings) have demonstrated excellent results for several combinations. For example, data from the National Joint Registry of England and Wales (NJR) showed that cemented stems with mixed polyethylene cups were associated with a lower risk for revision compared with their manufacturer-matched equivalents (Tucker et al. 2015).

For fixed combinations, different taper sizes used by the various manufacturers have made it difficult for surgeons to combine the stem and head junction properly, as the stems and head can vary in shape, metallurgy, roughness, inclination, and angle (Werner et al. 2015). Mixed components over the trunnion–taper junction in THAs with large head and hard-on-hard bearings may result in wear of the femoral head–neck interface (trunnionosis), which has been reported as an increasingly prevalent cause of failure (Mistry et al. 2016). In THAs with ceramic heads, a mismatch can result in a fractured femoral head component.

## Legal implications

The use of mixed components gives rise to legal implications from public and private law. One aspect of public law is that orthopedic implants have to be approved and marked Conformité Européene by an appropriate body before being allowed on the European market. This approval is given if the product meets the requirements of the Medical Devices Directive or its successor, the Medical Device Regulation, e.g., that the implant does not entail a safety risk (Directive 93/42/EEC 1993, Regulation (EU) 2017/745 2017). If a product is altered or a new product is designed by using several components that are not tested together, this approval might no longer be valid.

Implications may derive from private law too. The unauthorized mixing of components can give rise to a risk of liability toward patients, as liability could be imposed for (1) producing a defective product or (2) medical negligence.

## Product liability

Orthopedic surgeons who combine components from different manufacturers that are not designed, tested, or meant to be combined in compliance with the producers of the components bear a liability risk toward the patient. This risk derives from the European Product Liability Directive (85/374/EEC), which states that the producer of a product is liable for damages suffered by a patient if this product appears to be defective. This Directive is transposed into national law of all member states of the European Union and the European Free Trade Association. A healthcare provider who mixes components, such as a femoral head and stem from manufacturer A with a cup from manufacturer B, into a THA could qualify as a “manufacturer of a finished product” to whom the liability regime of the Directive applies (Gabrielczyk 2017).

## Defective product

In order for a producer (manufacturer or orthopedic surgeon) to be liable, the product has to be defective. This means that the product does not provide the safety that an individual is entitled to expect (article 6 of the Directive). Relevant in this respect is a recent English ruling that metal-on-metal (MoM) prostheses were not defective in terms of the entitled expectation of safety of such prostheses in 2002 (Colin Gee and others v. Depuy International Limited 2018). To determine whether a product provides the safety a person is entitled to expect, relevant circumstances are: the presentation of the product, the use to which the product could reasonably be expected to be put, and the time when the product was put into circulation. With regard to the latter: the defectiveness will be determined based on the state of knowledge and safety standards at the time it was put into circulation. The fact that a better product was subsequently put into circulation will not lead to the conclusion that the product in question must be considered defective. For orthopedic surgeons, this means that the state of knowledge at the time of insertion of the prothesis is important. In this respect it is relevant, for instance, that it was demonstrated in 2015 that the use of heads and stems from different manufacturers in mixed THAs leads to increased revision rates (Tucker et al. 2015).

Under certain circumstances, the producer can avoid the liability described above. Article 7 from the Directive sums up several defenses. For example, the producer will not be liable if it was impossible to know the risk that led to the defect because of a lack of knowledge. This refers to the objective scientific and technical knowledge available and accessible at that time, “including the most advanced level of such knowledge” (Commission of the European Communities v. United Kingdom of Great Britain and Northern Ireland 1997). As regards THAs the defense will most likely be unsuccessful if, at the time the THA was used by the surgeon, there was published scientific research available pointing out the risks of mixing components and materials.

## Medical negligence

Mixing of components could also result in liability of the healthcare provider if it qualifies as negligence. Liability for medical negligence is not regulated at the EU level, so regimes will vary per country. Generally, liability will require negligent behavior from the healthcare provider, meaning that he or she must have breached a standard of care (Cass 1936, HR 1990, BGH 1994, Bolam v. Friern HMC 2015). As opposed to the previous regime of strict liability of the producer, medical negligence generally requires that the healthcare provider commits a fault. Mixing of components might be considered negligent when it is unauthorized and discouraged by the manufacturer, untested by the orthopedic surgeon and unapproved according to public law—all the more in a primary situation, when reasonable alternatives are available and when medical publications have shown clinical risks. Whether or not the healthcare provider has acted negligently will be influenced by the communication of these risks to the patient and the receipt of the patient’s consent. And yet this might not be decisive due to the differences in knowledge and expertise between healthcare providers and patients. So, when mixing is a reasonable option, it is important to inform the patient about the use of mixed components, the benefits and potential risks, and reasonable alternatives, in order to gain the patient’s consent.

A search of case law in the United Kingdom (UK), Germany, and the Netherlands revealed that until now no orthopedic surgeon has ever been held responsible as the manufacturer of a finished product of mixed components. In the past one trial in the UK has been started but this has not resulted in a ruling in which the orthopedic surgeon was held responsible as the manufacturer of a finished product of mixed components.

## Conclusion

Mixing and matching in total hip arthroplasty is common practice worldwide. It is generally done in the interest of the patient, aiming to optimize the outcome of the treatment. We assessed the rules for mixed THAs based on European law, to create awareness of the legality. Despite evident medical benefits and similar or even superior revision rates compared with non-mixed THAs (Tucker et al. 2015, Peters et al. 2016, Taylor et al. 2018), from a legal perspective it is advisable to avoid mixing when reasonable alternatives are available, especially in primary arthroplasty. The unauthorized mixing of components can create a liability risk based on European and national law. An orthopedic surgeon who mixes components from different manufactures could qualify as a “manufacturer of a finished product” and may be held liable without fault if the product appears to be defective. However, to date, no orthopedic surgeon has been held legally responsible or ended up in a lawsuit for the use of mixed components, based on case law review in the United Kingdom, Germany, and the Netherlands. Although no search was done of case laws in other European countries we presume that the situation in these countries can be considered representative of the situation in Europe as a whole.

If a situation does require the use of mixed components, surgeons are best advised to (1) avoid mixing across the fixed articulation (i.e., use a head from the same manufacturer as the stem), (2) appropriately match sizes across the mobile articulation in hard-on-soft THAs (Tucker et al. 2015, Taylor et al. 2018), and (3) avoid mixing in hard-on-hard bearings. Surgeons are likewise advised to gain knowledge on the results of specific component combinations (e.g., based on arthroplasty registry results) and to explain the choices to the patient in order to receive his/her consent.
